# Anti-vascular endothelial growth factor in ophthalmology

**Published:** 2009

**Authors:** Rajesh Sinha, Sunil Choudhary, Subijoy Sinha, Nagender Vashisht, Chandrashekhar Kumar

Retinal hypoxia caused by any clinical condition results in the formation and release of vascular endothelial growth factor (VEGF). This can in turn result in formation of abnormal blood vessels at aberrant sites causing complications like intraocular hemorrhage and its sequelae. The strong supportive evidence from animal studies defined VEGF as an optimal therapeutic target for treatment of ocular diseases in which neovascularization leads to blindness.

Although there are many anti-VEGF described in the literature like Bevacizumab, Ranibizumab, Pegaptanib, Anecortave acetate, VEGF-trap, Squalamine lactate, Combretastatin A4 Prodrug, AdPEDF, SiRNA, Cand5, TG100801, the ones that have been most studied are Bevacizumab (Avastin; Genentech, Inc., CA) Ranibizumab (Lucentis; Genentech, Inc., CA), and Pegaptanib sodium (Macugen; Eyetech, Inc., NY).

Many studies have been performed to evaluate the role of anti-VEGF drugs in various clinical entities. We have reviewed some of the important studies in this article.

## Age related Macular Degeneration

### Bevacizumab

**Avery *et al.*** (Ophthalmology 2006;113(3):363-372) reported the short-term safety, biologic effect, and a possible mechanism of action of intravitreal bevacizumab (IVB) in patients with neovascular age-related macular degeneration (AMD). Eighty-one eyes of 79 patients with subfoveal neovascular AMD received IVB (1.25 mg) on a monthly basis until macular edema, subretinal fluid (SRF), and/or pigment epithelial detachment (PED) resolved. Most patients (55%) had a reduction of >10% of baseline retinal thickness at 1 week after the injection. At 4 weeks after injection, 30 of 81 eyes demonstrated complete resolution of retinal edema, SRF, and PEDs. Of the 51 eyes with 8 weeks’ follow-up, 25 had complete resolution of retinal thickening, SRF, and PEDs. At 4 and 8 weeks, mean visual acuity (VA) improved from 20/200 to 20/125 (*P*<0.0001). Short-term results suggested that IVB (1.25 mg) is well tolerated and associated with improvement in VA, decreased central macular thickness (CMT) by ocular coherence tomography (OCT), and reduction in angiographic leakage.

**Spaide *et al.*** (Retina 2006;26(4):383-90) described the short-term anatomical and visual acuity responses after IVB in patients with choroidal neovascularization (CNV) secondary to AMD in 251 eyes. The mean baseline VA was 20/184, and 175 (69.7%) eyes had inadequate response to alternate methods of treatment. At 1-month follow-up (data available for 244 patients), there was a significant decrease in CMT; the mean VA was 20/137 (*P* < 0.001), and 74 (30.3%) eyes had improvement in visual acuity as defined by halving of the visual angle.

**Costa *et al.*** (Invest Ophthalmol Vis Sci. 2006;47(10):4569-78) evaluated the safety of three dose regimen of IVB in 45 patients with AMD and subfoveal CNV. Compared with baseline, BCVA improved at week 1 (*P* = 0.001), week 6 (*P* < 0.001), and week 12 (*P* = 0.001). At week 12, the lesion area and CNV area were stable or decreased in 79.1% and 74.4% of patients, respectively.

**Dhalla *et al.*** (Retina 2006;26(9):988-93) evaluated the 7-month results for patients (n = 24) treated with combined photodynamic therapy (PDT) with verteporfin and IVB for juxtafoveal or subfoveal CNV. Sixteen eyes (67%) had improvement in VA by a mean of 2.04 Snellen lines.

**Costa *et al.*** (Graefes Arch Clin Exp Ophthalmol. 2007;245(9):1273-80) investigated the feasibility of combined IVB and PDT for CNV secondary to AMD in 11 patients with documented CNV progression after PDT treatment. The mean baseline logarithm of the minimum angle of resolution (logMAR) best corrected visual acuity (BCVA) on early treatment diabetic retinopathy study (ETDRS) chart was 1.031, which improved to 0.944, 0.924, 0.882, and 0.933 at 1, 2, 12 and 24 weeks respectively (*P*≤ 0.001), suggesting a possible synergistic effect of IVB and PDT.

**Cleary *et al.*** (Eye 2008;22(1):82-6) studied IVB in 112 eyes of 111 patients with neovascular AMD. They reported a statistically significant improvement in VA and reduction in CMT.

**Bashshur *et al.*** (Am J Ophthalmol. 2008;145(2):249-256) investigated the efficacy of IVB in 60 eyes with subfoveal CNV attributable to AMD. In the initial treatment phase, IVB (2.5 mg/0.1 ml) was given at baseline, and then two additional monthly injections were given if the macula was not dry on OCT. The criteria for re-injection after the induction phase were presence of new fluid in the macula, increased CMT by at least 100μ, loss of at least five letters of vision with increased fluid in the macula, new classic CNV or new macular hemorrhages. Fifty-one patients (51 eyes) completed the 12 months follow up. Mean visual acuity improved from 45.7 letters at baseline to 53.1 letters at 12 months (*P* = 0.004), and 47 eyes (92.2%) lost <15 letters. Mean CRT decreased from 327.4μ at baseline to 227.8μ at 12 months (*P*< 0.001). A mean of 3.4 injections were given over the course of the study, and no ocular or systemic side-effects were noted.

**Ghazi *et al.*** (Retina 2008;28(5):689-95) performed IVB treatment in 13 patients of retinal angiomatous proliferation (RAP) in neovascular AMD and reported it to be a viable treatment option. The average BCVA improved from 20/203 at baseline to 20/113 at 12 weeks (*P* = 0.001). The average CMT improved from 369μ at baseline to 315μ (*P* = 0.020) at 12 weeks.

**Furino *et al.*** (Acta Ophthalmol. 2008; 6) evaluated the efficacy of multiple IVB injections for treatment-naive subfoveal occult CNV in AMD in twelve eyes of 12 patients They concluded that multiple IVB injections are well tolerated and associated with significant improvements in BCVA and decreased CMT by OCT in most patients with treatment-naive occult CNV.

**Arevalo *et al.*** (Retina 2008;28(10):1387-94) performed IVB in 63 eyes with subfoveal CNV and concluded that primary IVB at doses of 1.25 mg or 2.5 mg improves BCVA, and reduces macular thickness in subfoveal CNV.

### Ranibizumab

**Rosenfeld *et al.*** (Ophthalmology 2006;113(4):623) performed multiple intravitreal injections of ranibizumab (up to 2mg), in patients with primary or recurrent subfoveal CNV secondary to AMD. Treatment regimens consisted of 5, 7, or 9 intravitreal ranibizumab (IVR) at 2- or 4-week intervals for 16 weeks, with escalating doses ranging from 0.3 to 2.0 mg. Overall, median and mean BCVA in the study eyes improved by day 140 in all 3 groups. Only 3 of the 27 patients lost significant vision. There was no significant lesion growth, and a decrease in area of leakage from CNV was detected through day 140.

**Rosenfeld *et al.*** [MARINA Study Group] (N Engl J Med 2006;355(14):1419-31) performed a multicenter, 2-year, double-blind, sham-controlled study, in which 716 patients with AMD with either minimally classic or occult (with no classic lesions) CNV were randomly assigned to receive 24 monthly IVR (either 0.3 mg or 0.5 mg) or sham injections. At 12 months, 94.5% of the group given 0.3 mg of ranibizumab and 94.6% of those given 0.5 mg lost fewer than 15 letters, as compared with 62.2% of patients receiving sham injections (*P*<0.001 for both comparisons). Visual acuity improved by 15 or more letters in 24.8% of the 0.3-mg group and 33.8% of the 0.5-mg group, as compared with 5.0% of the sham-injection group (*P*<0.001 for both doses). The benefit in visual acuity was maintained at 24 months. They concluded that IVR for 2 years prevented vision loss and improved mean VA, with low rates of serious adverse events, in patients with minimally classic or occult CNV secondary to AMD.

**Brown *et al.*** [ANCHOR Study Group] (N Engl J Med. 2006 5;355(14):1432-44) performed a 2-year, multicenter, double-blind study, in which they randomly assigned patients in a 1:1:1 ratio to receive monthly IVR (0.3 mg or 0.5 mg) plus sham verteporfin therapy or monthly sham injections plus active verteporfin therapy. Of the 423 patients enrolled, 94.3% of those given 0.3 mg of ranibizumab and 96.4% of those given 0.5 mg lost fewer than 15 letters, as compared with 64.3% of those in the verteporfin group (*P*<0.001 for each comparison). Visual acuity improved by 15 letters or more in 35.7% of the 0.3-mg group and 40.3% of the 0.5-mg group, as compared with 5.6% of the verteporfin group (*P*<0.001 for each comparison). Mean visual acuity increased by 8.5 letters in the 0.3-mg group and 11.3 letters in the 0.5-mg group, as compared with a decrease of 9.5 letters in the verteporfin group (*P*<0.001 for each comparison). They concluded that ranibizumab was superior to verteporfin for the treatment of predominantly classic neovascular AMD, with fewer ocular adverse events.

**Heier *et al.*** [FOCUS Study Group] (Arch Ophthalmol. 2006;124(11):1532-42) investigated the safety and efficacy of IVR combined with verteporfin PDT in patients with predominantly classic neovascular AMD. They concluded that IVR combined with PDT was more efficacious than PDT alone for treating neovascular AMD.

**Rothenbuehler *et al.*** (Am J Ophthalmol. 2009;147(5):831-7) in their study reported not only prevention of vision loss but also improvement in BCVA and reduction of CMT with IVR (0.5 mg) on a variable-dosing regimen over 24 months in 138 eyes with AMD.

**Lalwani *et al.*** (Am J Ophthalmol. 2009;17) assessed the long-term efficacy of a variable-dosing regimen with ranibizumab in the Prospective Optical Coherence Tomography (OCT) Imaging of Patients with Neovascular AMD Treated with intra Ocular Ranibizumab (PrONTO) Study. In this open-label, prospective, single-center, uncontrolled clinical study, subfoveal neovascular AMD patients with a CMT of at least 300μ on OCT were enrolled to receive 3 consecutive monthly IVR (0.5 mg). Retreatment with ranibizumab was performed if indicated. Forty patients were enrolled and 37 completed the 2-year study. At month 24, the mean visual acuity (VA) improved by 11.1 letters (*P* < 0.001) and the OCT-CMT decreased by 212μ (*P* < 0.001). The VA improved by 15 letters or more in 43% of patients. These VA and OCT outcomes were achieved with an average of 9.9 injections over 24 months. The PrONTO Study using an OCT-guided variable-dosing regimen with IVR reported VA outcomes comparable with the outcomes from the phase III clinical studies, but fewer intravitreal injections were required.

**Konstantinidis *et al.*** (Graefes Arch Clin Exp Ophthalmol. 2009 Apr 29) evaluated the functional and anatomic outcome after IVR treatment in 31 eyes of 31 patients with RAP. The mean logMAR BCVA improvement (mean number of injections=5) and reduction in CMT from the baseline was highly significant (*P* < 0.0001).

### Pegaptanib sodium

**Tano *et al.*** (Nippon Ganka Gakkai Zasshi. 2008;112(7):590-600) randomly assigned ninety-five Japanese patients with subfoveal neovascular AMD in a double-masked manner to receive 0.3 or 1 mg of pegaptanib sodium every 6 weeks over a 48-week period and concluded that intravitreal injection of pegaptanib every 6 weeks produces clinically significant stabilization of visual acuity for one year in more than 70% of patients with AMD, with good treatment compliance.

**Chakravarthy *et al.*** [VEGF Inhibition Study in Ocular Neovascularization (V.I.S.I.O.N.) Clinical Trial Group] (Ophthalmology2006;113(9):1508) evaluated the efficacy of a second year of pegaptanib sodium therapy in patients with neovascular AMD. Continuing visual benefit was observed in patients who were randomized to receive therapy with pegaptanib in year 2 of the V.I.S.I.O.N. trials when compared with 2 years’ usual care or cessation of therapy at year 1.

**Maier *et al.*** (Klin Monatsbl Augenheilkd. 2008;225(6):582-7) reported their experience with pegaptanib for the treatment of occult or minimally classic CNV due to AMD and concluded that intravitreal Pegaptanib was safe and effective with stabilisation in all the assessed parameters.

**Wykrota *et al.*** (Klin Oczna. 2007;109(10-12):394-401) evaluated the efficacy of intravitreal pegaptanib sodium in CNV due to AMD in 38 eyes of 38 treatment-naive patients. Intravitreal injections were performed every 6 weeks at the discretion of the treating ophthalmologist. They concluded that Pegaptanib sodium effectively preserved vision in approximately 70% of patients with wet AMD over a period of 1-year.

**Ehlers *et al.*** (Ophthalmic Surg Lasers Imaging 2007;38(5):371-7) in a similar study reported that Pegaptanib therapy resulted in a 2.9 line loss in patients when all lesions were considered. Small lesions responded favorably, with 15% of patients gaining more than 3 lines of VA. Larger lesions had an increased risk of progression and poor visual outcome.

**Quiram *et al.*** (Retina 2007;27(7):851-6) retrospectively reviewed data of 90 patients with newly diagnosed wet AMD (80% occult, 13% minimally classic, and 7% predominantly classic) in which pegaptanib was used as primary therapy. Lesion sizes were 50% ≤4 disc areas (DA) and 50% >4 DA. The mean follow-up was 9.1±2.0 months. Gain of ≥3 lines of vision occurred in 20% of patients, stabilization of vision (prevention of three lines of vision loss) occurred in 70% of patients, and loss of ≥3 lines of vision occurred in 10% of patients, resulting in a 90% response rate. In the patients who gained ≥3 lines of vision, the average number of injections was 3.5. One case of endophthalmitis was recognized.

### Combined Bevacizumab and Pegaptanib

**Hughes *et al.*** (Ophthalmic Surg Lasers Imaging 2006;37(6):446-54) combined nonselective (bevacizumab) and selective VEGF blockade (pegaptanib) to achieve clinical benefit with minimal risks in the treatment of neovascular AMD and reported a favourable outcome.

## Diabetic Retinopathy

### Bevacizumab

#### A. Diabetic Macular Edema (DME)

**Scott *et al.*** [Diabetic Retinopathy Clinical Research Network] (Ophthalmology 2007;114(10): 1860-7) conducted a study in which 121 eyes of 121 subjects with DME and Snellen acuity equivalent ranging from 20/32 to 20/320 were divided randomly into 5 groups: (A) focal photocoagulation at baseline (n=19), (B) intravitreal injection of 1.25 mg of bevacizumab at baseline and at 6 weeks (n=22), (C) intravitreal injection of 2.5 mg of bevacizumab at baseline and at 6 weeks (n=24), (D) intravitreal injection of 1.25 mg of bevacizumab at baseline and sham injection at 6 weeks (n=22), or (E) intravitreal injection of 1.25 mg of bevacizumab at baseline and at 6 weeks with photocoagulation at 3 weeks (n=22). Compared with group A, groups B and C had a greater reduction in CST at 3 weeks and about 1 line better median VA at 12 weeks. There were no meaningful differences between groups B and C in CST reduction or VA improvement. A CST reduction > 11% (reliability limit) was present at 3 weeks in 43% of bevacizumab-treated eyes and 28% of eyes treated with laser alone, and at 6 weeks in 37% and 50% eyes, respectively. They concluded that combining focal photocoagulation with bevacizumab resulted in no apparent short-term benefit or adverse outcomes.

**Arevalo *et al.*** [Pan-American Collaborative Retina Study Group] (Ophthalmology 2007; 114(4):743-50) studied IVB in patients with DME and reported that 20.5% of eyes needed a second injection at a mean of 13.8 weeks, and 7.7% needed a third injection at a mean of 11.5 weeks. The mean baseline BCVA was 0.87, and the final mean BCVA was 0.6 (*P*<0.0001). Final BCVA analysis by subgroups demonstrated that 41.1% eyes remained stable, 55.1% improved ≥2 lines of BCVA, and 3.8% decreased ≥2 lines of BCVA. Mean CMT at baseline by OCT was 387.0±182.8μ that decreased to a mean of 275.7±108.3 at the end of follow-up (*P*<0.0001). They concluded that primary IVB at doses of 1.25 to 2.5 mg seem to provide stability or improvement in VA, OCT, and fundus fluorescein angiography in DME at 6 months.

**Kook *et al.*** (Retina 2008;28(8):1053-60) evaluated the long-term efficacy of bevacizumab for the treatment of chronic diffuse DME in 126 eyes after various previous treatments. At baseline, mean VA on ETDRS chart was 40.3 letters and mean CMT on OCT was 463μ. Throughout follow-up, VA changes were not significant with a mean change of −1.6 ETDRS letters at 6 months, but significant with +5.1 ETDRS letters at 12 months. Mean CMT decreased to 374μ at 6 months (*P* < 0.001) and to 357μ at 12 months (*P* < 0.001).

**Bonini-Filho** (Am J Ophthalmol. 2009;Mar 25. Epub ahead of print) noted favorable changes in BCVA and in CMT through 1 year following IVB in eyes with DME and severe capillary loss.

**Shimura *et al.*** (Am J Ophthalmol. 2008;145(5):854-61) compared the effect of IVB (1.25mg) and intravitreal triamcinolone acetonide [IVTA] (4.0mg) on persistent diffuse DME in 28 eyes of 14 patients. They found that IVTA showed better results in improving BCVA and reducing DME. However, both groups showed recurrence of macular edema with time.

**Faghihi *et al.*** (Eur J Ophthalmol. 2008;18(6):941-8) compared combined IVTA and IVB (n=41) with standard macular laser photocoagulation [MPC] (n=47) and IVB alone (n=42) in the management of DME in 130 eyes of 110 patients. They found that at 6 weeks, all the three groups showed significant reduction in CMT but the reductions for IVB and IVB+IVT were significantly more than MPC (*P*<0.001). However, at week 16, the response was not stable for IVB (*P*<0.001), but IVB+IVT maintained its superior status to MPC (*P*<0.001) and the VA were essentially unchanged in MPC and IVB groups and improvement for IVB+IVT was marginal.

**Paccola *et al.*** [IBEME study] (Br J Ophthalmol. 2008;92(1):76-80) compared single IVTA (4mg) versus IVB (0.06ml) injections in treating refractory DME. The CMT reduced significantly in the IVTA group compared with the IVB group at weeks 4, 8, 12 and 24 (*P* <0.05). The BCVA was significantly higher at weeks 8 and 12 in IVTA group compared to IVB group.

**Yanyali *et al.*** (Am J Ophthalmol. 2007;144(1):124-6) in their study reported that mean CMT and VA was same even after multiple injections of IVB for DME in previously vitrectomized eyes.

#### B. Proliferative Diabetic Retinopathy (PDR)

**Cho *et al.*** (Retina 2009;29(4):516-22) evaluated the efficacy and safety of IVB as an adjunctive treatment to pan retinal photocoagulation (PRP) for high-risk PDR with or without clinically significant macular edema (CSME). They concluded that the combination of IVB with PRP can be effective in treatment of high risk PDR, especially if there is no CSME.

**Moradian *et al.*** (Graefes Arch Clin Exp Ophthalmol. 2008;246(12):1699-705) evaluated the efficacy of IVB in eyes with active and progressive PDR. They concluded that IVB has significant therapeutic effect on eyes with active, progressive PDR; the treatment causes a significant amount of vitreous haemorrhage resolution and new vessel regression. However, this procedure may increase the risk of TRD in eyes with fibrous proliferation.

**Cernak *et al.*** (Cesk Slov Oftalmol. 2008;64(6):234-6) gave IVB injection (1.25 mg) in three eyes with PDR and neovascularization of iris (NVI) and reported disappearance of NVI and normalization of intraocular pressure (IOP) with no side effects.

**ReJorge *et al.*** (Retina 2006;26(9):1006-13) in their study found that post-IVB there was short-term reduction of fluorescein leakage from persistent active neovascularization without loss of vision in patients with diabetic retinopathy.

**Shih *et al.*** (Graefes Arch Clin Exp Ophthalmol. 2008;246(11):1547-51) reported that IVB and C3F8 gas may be an effective method for treating acute diabetic premacular hemorrhage with active fibrovascular proliferation.

**Arevalo *et al.*** (Br J Ophthalmol. 2008 Feb;92(2):213-6) observed that TRD developed in patients (11/211 eyes) with severe PDR following IVB within 2 weeks.

### Pegaptanib in Diabetic Retinopathy

**Adamis *et al.*** (Ophthalmology 2006;113(1):23-8) studied effects of intravitreal pegaptanib on retinal neovascularization. Most subjects with retinal neovascularization at baseline showed regression of neovascularization by week 36.

### Ranibizumab in DME

**Chun *et al.*** (Ophthalmology 2006; 113(10):1706-12) evaluated the biologic activity of multiple IVR injections in patients with center-involving CSME and reported that ranibizumab therapy has the potential to maintain or improve BCVA and reduce CMT in these eyes.

## Vascular Occlusion

### Bevacizumab

**Iturralde *et al.*** (Retina 2006;26(3):279-84) reported the short term anatomic and visual acuity response after IVB (mean: 2.8 injections) in patients with macular edema due to central retinal vein occlusion (CRVO) in 16 eyes. The mean CMT at baseline was 887μ and decreased to a mean of 372μ at month 1 (*P* < 0.001). The mean baseline acuity was 20/600 (logMAR = 1.48) and the mean acuity at 1month was 20/200 (logMAR = 1.05) (*P* = 0.001) and at 3 months it was 20/138 (logMAR = 0.84) (*P* < 0.001).

**Costa *et al.*** (Retina 2007;27(2):141-9) in their study reported that IVB injections of 2.0 mg at 12-week intervals were well tolerated and were associated with short-term BCVA stabilization or improvement and favorable macular changes in all patients with ischemic CRVO.

**Rabena *et al.*** (Retina 2007;27(4):419-25) reported good efficacy of IVB in patients with macular edema (ME) secondary to branch retinal vein occlusion (BRVO). There was significant reduction of CMT and improvement of VA with lack of serious side effects.

**Priglinger *et al.*** (Retina 2007;27(8):1004-12) performed a study in which 46 patients of CRVO received repeated IVB (1.25 mg). Mean visual acuity improved from 20/250 at baseline to 20/80 at the 6-month follow-up (*P* < 0.001). There was a mean gain of 13.9 ±14.4 letters (ETDRS) at 6 months. The mean CMT decreased from 535±148μ at baseline to 323±116μ at 6 months. The gain in VA was similar in both ischemic and non-ischemic types.

**Rensch *et al.*** (Acta Ophthalmol. 2009;87(1):77-81) evaluated the effect of early IVB for the treatment of macular oedema caused by non-ischaemic CRVO in 25 eyes. They gave three IVB injections (1.5 mg) with an interval of 6 weeks between injections. The mean duration of CRVO prior to the first injection was 4.2±3.6 days. The mean VA improved significantly from 0.97±0.40 at baseline to 0.70±0.42 (*P* = 0.007) at 1 month, 0.69±0.46 (*P* = 0.006) at 3 months and 0.69 ±0.52 (*P* = 0.015) at 6 months after the first injection. Mean CMT decreased significantly from 530±152μ at baseline to 347±127μ (*P* < 0.001) at 1 month, 370 ±165μ (*P* < 0.001) 3 months and 346 ±129μ (*P* < 0.001) 6 months (*P* < 0.001) after the first injection.

### Ranibizumab

**Spaide *et al.*** (Am J Ophthalmol. 2009;147(2):298-306) evaluated intravitreal injection of ranibizumab for the treatment of macular edema due to CRVO in 20 eyes. There was a significant increase in VA and a significant reduction in CMT at 1 year.

### Retinopathy of Prematurity

**Mintz-Hittner and Kuffel.** (Retina 2008;28(6):831-8) gave bilateral IVB injections in moderate and severe stage 3 retinopathy of prematurity (ROP) in zone I or posterior zone II. Eleven infants weighing from 515g to 1,015g at birth with gestational ages from 23 to 28 weeks received IVB (0.625 mg [0.025 ml]) at 9.0 weeks to 15.0 weeks of age and never had laser therapy. All 22 eyes were treated successfully (no retinal detachment, macular ectopia, high myopia, anisometropia, or other ocular abnormalities) with only 1 injection. They concluded that IVB was safe and effective in treating stage 3 ROP in zone I and posterior zone II.

**Kusaka *et al.*** (Br J Ophthalmol. 2008;92(11):1450-5) reported that IVB seems to be associated with reduced neovascularisation without apparent ocular or systemic adverse effects, and is thus beneficial for treating severe ROP that is refractory to conventional laser therapy.

### Neovascular Glaucoma

**Iliev *et al.*** (Am J Ophthalmol. 2006;142(6):1054-6) studied IVB (1.25 mg/0.05 ml) in 6 patients with neovascular glaucoma (NVG) and a pronounced NVI and neovascularisation of angle (NVA). There was marked regression of neovascularization, relief of symptoms with a substantial reduction in IOP in 3 eyes within 48 hours; in the other 3 eyes, adjuvant cyclophotocoagulation was needed.

**Gheith *et al.*** (J Ocul Pharmacol Ther. 2007;23(5):487-91) retrospectively reviewed 6 consecutive cases of NVG who received 1.25 mg (0.05 cc) of IVB followed by PRP 1 week later and followed-up for 3-19 months. In all cases, there was a complete regression of NVI; however, 2 eyes showed a recurrence of neovascularization and by repeat injection and additional PRP, there was complete resolution of neovascularization.

**Yuzbasioglu *et al.*** (J Ocul Pharmacol Ther.2009;25(3):259-64) reported that NVI and NVA resolved completely after 36 hours after simultaneous intravitreal and intracameral injection of 1.25 mg bevacizumab in all 15 cases of NVG. The IOP reduced to less than 22 mmHg in six cases without any medication.

**Moraczewski *et al.*** (Br J Ophthalmol. 2009;93(5):589-93) also reported in their study that IVB is a useful adjunct in resolving NVI and NVG. However, these eyes must be monitored closely after initial injection of IVB, as many may still require surgery or repeat injections to lower IOP.

**Wasik *et al.*** (Optometry 2009;80(5):243-8) also reported in their study that IVB in conjunction with PRP could be a treatment option for NVG.

**Beutel *et al.*** (Acta Ophthalmol. 2008;Sep 20.Epub ahead of print) evaluated the long term effects of IVB in patients with NVG and found that complete regression of NVI was detectable in 20% eyes, incomplete regression in 35%, stabilization in 30%, and an increase in 10% of eyes. There was good control of IOP, however additional treatments were required.

## Anti-VEGF Treatment in Corneal and Ocular Surface Disorders

### Reduction of Corneal Vascularisation

**Dastjerdi *et al.*** (Arch Ophthalmol. 2009;127(4):381-9) studied the safety and efficacy of topical bevacizumab 1.0% for 3 weeks in the treatment of corneal neovascularization (NV) in 10 eyes. At 24 weeks, the mean (standard deviation) reductions in corneal NV were 47.1% (36.7%) for neovascular area (*P*=.001), 54.1% (28.1%) for vessel caliber (*P*<.001), and 12.2% (42.0%) for invasion area. Topical bevacizumab was well tolerated with no adverse events.

**Kim *et al.*** (Ophthalmology 2008;115(6):e33-8) examined the effect of topical bevacizumab (1.25%) twice daily on corneal NV in 10 eyes over 3 months. There was a decrease in corneal NV in 7 of 10 eyes within 1 month. Epitheliopathy (epithelial defect, epithelial erosion) was observed in 6 of 10 eyes, 1 resulting in corneal thinning during the second month of treatment.

**Bahar** (Cornea 2008;27(2):142-7) reported that subconjunctival bevacizumab [SCB] (2.5 mg/0.1 ml) is well tolerated and associated with a partial regression of corneal neovascularization.

## Chemical Injury

**Oh *et al.*** (Curr Eye Res. 2009;34(2):85-91) reported that SCB showed a borderline reduction in corneal NV and a significant reduction of inflammatory cells in an experimental rat model.

Yoeruek *et al.* (Acta Ophthalmol. 2008;86(3):322-8) evaluated bevacizumab eye drops (25 mg/ml) administered five times daily in corneal NV induced experimentally by alkali burn and reported a significant reduction in corneal damage by its antiangiogenic and antifibrotic effect.

### Pterygium

**Wu *et al.*** (Cornea 2009;28(1):103-4) reported regression of pterygium with bevacizumab eye drops administered 4 times daily for 3 weeks with no recurrence till 6 months.

**Teng *et al.*** (Cornea 2009;28(4):468-70) reported a temporary (up to 7 weeks) regression of hyperemia in primary pterygium with SCB (1.25 mg/0.05 ml).

## Corneal Grafts

**Gerten** (Cornea 2008;27(10):1195-9) treated two eyes with corneal NV with preoperative argon laser and SCB and corneal transplantation was performed 6 weeks later along with intraocular injection of bevacizumab. A marked reduction of corneal NV was observed in both the eyes and no recurrence of NV spreading into the graft was observed with clear grafts till 6 months.

**Qian *et al.*** (Cornea 2008;27(9):1090-2) reported the effect of superficial keratectomy combined with SCB injection (2.5 mg/0.1 ml) in 2 cases of corneal NV; one due to sclerokeratitis secondary to rheumatoid arthritis and the other due to Terrien's marginal degeneration. Corneal NV regressed with the surgical removal and showed no signs of recurrence till 3 months.

**Doctor *et al.*** (Cornea 2008;27(9):992-5) also reported regression of corneal NV with SCB (1-3 injections; 2.5 mg) in 8 eyes with corneal NV secondary to ocular surface inflammatory diseases.

### Herpetic stromal keratitis

**Carrasco** (Cornea 2008;27(6):743-5) treated an 81-year-old woman with corneal NV secondary to herpetic stromal keratitis with SCB and reported a dramatic regression of corneal vessels 1 week after the injection with no recurrence till 3 months.

## Steven Johnson syndrome

**Uy *et al.*** (Cornea 2008;27(1):70-3) evaluated the efficacy and safety of topical bevacizumab (25 mg/ml) 4 times daily for a period of 3 months on ocular surface NV in 2 patients (3 eyes) with Stevens-Johnson syndrome. At the end of the study period, visual acuity improved in all 3 eyes with decreased ocular surface NV, corneal opacification, and conjunctival injection.

## Safety profile of Anti-VEGF

**Bock *et al.*** (Invest Ophthalmol Vis Sci. 2009;50(5):2095-102) analyzed the safety profile of bevacizumab eye drops (5 mg/ml) and an antimurine VEGF-A antibody (250 μg/ml) applied to normal murine corneas five times a day for 7 and 14 days. There were no obvious corneal morphologic changes and no significant impact on wound healing after corneal epithelial injury.

**Ladas *et al.*** (Retina 2009;29(3):313-8) compared the safety of repeated IVB and IVR injections in 450 patients (2,000 injections; 1,275 IVB and 725 IVR) over 2 years. The commonest adverse events were injection-site redness (64.75%) [procedure related] and uveitis (1.90%) [drug related]. Multiple injections of both drugs were well tolerated and safe and were comparable.

**Heier *et al.*** (Ophthalmology 2006;113(4):633) also reported a good safety profile of multiple injections of ranibizumab in 64 patients with subfoveal neovascular AMD.

### Combined with Surgical Procedure

#### Phacoemulsification

**Cheema *et al.*** (J Cataract Refract Surg. 2009;35(1):18-25) evaluated the role of IVB, when injected at the time of cataract surgery on the postoperative progression of diabetic retinopathy (DR) and diabetic maculopathy (DM). They noted progression of DR in 45.45% of eyes in the control group and 11.42% of eyes in the intervention group (*P* = 0.002) and progression of DM in 51.51% of eyes in the control group and 5.71% of eyes in the intervention group (*P* = 0.0001).

## Pars plana vitrectomy (PPV)

**Figueroa *et al.*** (Curr Diabetes Rev. 2009;5(1):52-6) in their study reported that pre-operative injection of anti-VEGF reduces neovascularization and adherence of fibrovascular complex to the retina. This simplifies delamination and reduces intraoperative bleeding. It also prevents re-bleed, however, PPV must be performed within a week after injection to minimize risk of TRD.

**Rizzo *et al.*** (Graefes Arch Clin Exp Ophthalmol. 2008;246(6):837-42) in their study reported that IVB administered prior to PPV in PDR was well tolerated and reduced active neovascularization, thus facilitating PPV.

**Yeoh *et al.*** (Clin Experiment Ophthalmol. 2008;36(5):449-54) in their study reported that IVB used as an adjunct to PPV is useful in diabetic eyes with TRD of short duration in which there is still active neovascularization.

**Oshima *et al.*** (Ophthalmology 2009;116(5):927-38) investigated the feasibility and efficacy of microincision vitrectomy surgery combined with IVB for treating TRD secondary to severe PDR. They noticed that anatomical attachment rates of this procedure are comparable with conventional 20-gauge PPV. In addition, this technique shortens the surgical time with fewer intraoperative complications and favorable visual recovery.

**da R Lucena *et al.*** (Br J Ophthalmol. 2009;93(5):688-91) found that preoperative IVB was associated with reduced intraocular bleeding during 23-gauge PPV for diabetic macular TRD.

**Romano *et al.*** (Eye 2008 Nov 28. Epub ahead of print) suggested that IVB injection few days before planned PPV facilitates the surgery and reduces the risk of re-bleed.

## Glaucoma Surgery

**Cornish *et al.*** (Eye 2009;23(4):979-81) performed trabeculectomy (Trab) with mitomycin C (MMC) for NVG in combination with IVB. They observed that NVI resolved within 48 hours with good control of IOP (between 10-15 mmHg) at 6 months.

**Kitnarong *et al.*** (Adv. Ther. 2008;25(5):438-43) evaluated the surgical outcome of Trab with MMC in NVG after an adjunctive treatment with IVB. They observed absolute regression of NVI within 1 week after IVB in four patients, while in two patients, NVI regressed but still persisted. The mean IOP decreased from 39.8 mmHg pre-operatively to 7.5 mmHg on the first postoperative day. During the mean follow up of 24.7 weeks, five patients had controlled IOP (range, 2-16 mmHg) without antiglaucoma medication.

